# Imaging observations of a schwannoma of low malignant potential in the anterior abdominal wall: A case report

**DOI:** 10.3892/ol.2014.2305

**Published:** 2014-07-02

**Authors:** YONGKANG LIU, XIAO CHEN, TIANYAO WANG, ZHONGQIU WANG

**Affiliations:** 1 Department of Radiology, Affiliated Hospital of Nanjing University of Traditional Chinese Medicine, Nanjing, Jiansu 210029, P.R. China; 2 Department of Radiology, Shanghai East Hospital, Tongji University School of Medicine, Shanghai 200120, P.R. China

**Keywords:** schwannoma, abdominal wall, computed tomography, ultrasound

## Abstract

Neurilemmoma, also known as schwannoma, is an uncommon benign neoplasm that is most commonly found in the trunk and head and neck regions. The present study reports the case of a 67-year-old female with schwannoma localized in the anterior abdominal wall and analyzes the ultrasound and computed tomography (CT) imaging observations of the schwannoma. A dynamic time-intensity curve was also recorded in the study. A well-defined, elliptic low echo level, heterogeneous mass was observed during ultrasound examination. The CT scan revealed a solid, heterogeneous, low-density mass in the abdominal wall. Contrast-enhanced scans showed a heterogeneously enhanced mass during the arterial and venous phase. Centripetal fill-in was demonstrated and the mass was markedly, homogenously enhanced relative to the muscles during the delayed phase. Peak enhancement was observed during the venous phase and then slowly declined. However, the mass was hyperattenuated during the delayed phase. The lesion was completely excised and no evidence of recurrence has been identified during the 3 months of follow-up. The present study suggested that a diagnosis of schwannoma should be considered for certain patients with masses in the abdominal wall. Peripheral enhancement during the arterial and venous phases and homogeneous enhancement in the delayed phase are the significant imaging findings of a schwannoma.

## Introduction

Neurilemmoma, also known as schwannoma, is an uncommon benign neoplasm originating from the Schwann cells in the peripheral nerve sheaths (1,2). The majority of the tumors commonly localize in the trunk, head and extremities (3). However, occurrence in the anterior abdominal wall is extremely rare. The present study reports the case of a 67-year-old female with a schwannoma of low malignant potential in the right anterior abdominal wall and analyzes the main ultrasound and computed tomography (CT) imaging observations. This study highlights the clinical features and imaging findings of schwannoma in the abdominal wall, and may aid in the future diagnosis and differential diagnosis of this condition. Patient provided written informed consent.

## Case report

### Patient and clinical data

A 67-year-old female was admitted to the Affiliated Hospital of Nanjing University of Traditional Chinese Medicine (Nanjing, China) complaining of a disconcerting mass in the right anterior abdominal wall. A small mass had incidentally been found 10 years ago, with no restriction of movement present in the right abdominal wall. As the patient did not exhibit any evident uncomfortable symptoms, not enough attention was drawn to the lesion. Over the year prior to the present hospital admittance, the lump gradually increased in size and the patient felt soreness and suffered untimely gas pains. To acquire a diagnosis and treatment, the patient was referred to the outpatient clinic of the the Affiliated Hospital of Nanjing University of Traditional Chinese Medicine. There was no history of anergy, fever, anorexia, weight loss, trauma or surgery, and there was no family history of a similar complaint.

A physical examination showed a 6×4-cm mass protruding through the right anterior abdominal wall. The mass was firm, non-tender and not fixed to the skin of the abdominal wall. All routine laboratory tests were within the normal ranges.

### Ultrasound and CT imaging findings

**Figure 1:**
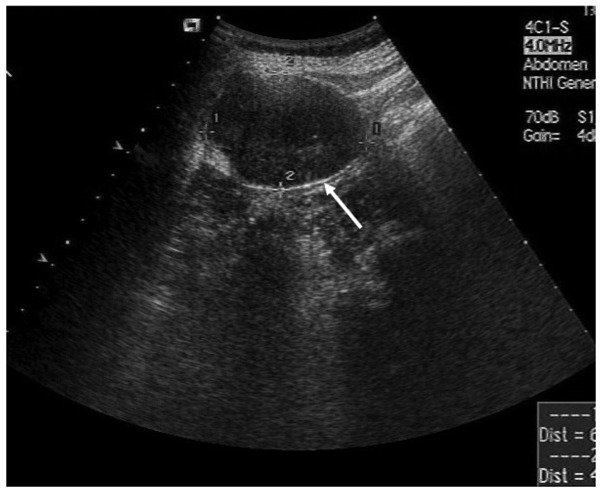
B-mode ultrasound showing the well-encapsulated mass in the anterior abdominal wall (arrow).

**Figure 2:**
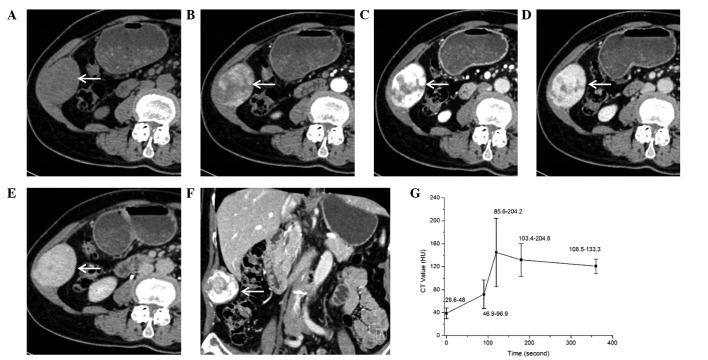
Computed tomography (CT) imaging findings of the schwannoma. (A) Conventional CT scan revealing an oval mass (white arrow) in the right lower abdominal wall. Contrast-enhanced CT scan showing a gradually- and heterogeneously-enhanced lesion with high density in (B) the arterial phase, (C) the venous phase and (D and E) the delay phase at different times. (F) Coronal image showing that the mass is in the abdominal wall. (G) The time-intensity curve displaying the characteristics of enhancement following agent injection.

An abdominal ultrasound examination showed a well-defined, elliptic, low echo level, heterogeneous mass just beneath the abdominal skin (Fig. 1). The diameter of the tumor was 5.6 cm. A CT scan without contrast enhancement revealed a solid, homogeneous mass with a low density relative to the muscle in the abdominal wall (Fig. 2A). Contrast-enhanced scans showed a evident gradually- and heterogeneously-enhanced lesion during the arterial (Fig. 2B) and venous (Fig. 2C) phases. Centripetal fill-in was demonstrated and the mass was markedly, homogenously-enhanced relative to the muscles (Fig. 2D and E) during the balanced and delayed phases. The coronal CT image also clearly showed that the mass was localized in the abdominal wall (Fig. 2F). The tumor showed marked enhancement on contrast-enhanced CT. The time-intensity curve following contrast agent injection is shown in Fig. 2G. Peak enhancement was observed during the venous phase (120 sec after contrast agent injection) and then slowly declined. However, the mass was hyperattenuated during the delayed phase (360 sec after contrast agent injection). No cysts, calcification or necrosis were found in the patient even though the mass was large. A radiological diagnosis of a paraganglioma or a vascular genesis tumor was considered.

### Surgery and histopathological examination

**Figure 3:**
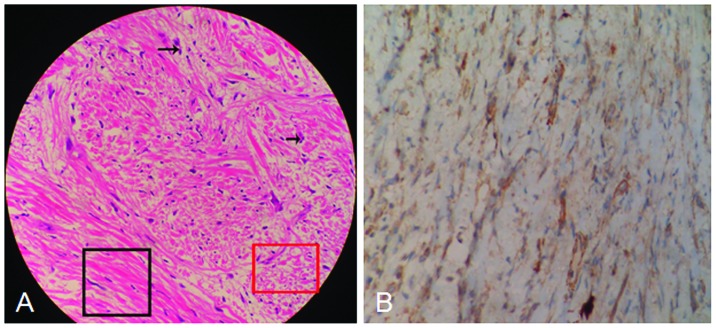
(A) Histopathological and (B) immunochemical appearance of the schwannoma (hematoxylin and eosin stainning; magnification, ×40). (A) Abundant spindle-shaped cells localized in the Antoni type A component area (black frame) and the Antoni type B component area (red frame). Sporadic karyokinesis (arrow) was occasionally observed (magnification, ×40). (B) Immunohistological assay showing cells negative for S-100 staining.

The lesion was completely excised and a frozen section of the specimen was sent for histological examination. Histological examination (Fig. 3A) showed that the tumor was composed of abundant spindle-shaped cells, which locally invaded the surrounding fat tissues. Karyokinesis of the tumor cells could occasionally be observed. A primary diagnosis of a spindle cell tumor and neurofibroma was made. Immunohistochemical examination showed that the specimen was negative for S-100 (Fig. 3B). Based on the imaging findings and histopathological results, a final diagnosis of a schwannoma of low malignant potential was made. The patient was discharged 10 days after the surgery and no further treatment was required. No evidence of recurrence was found during the 3-month follow-up.

## Discussion

Schwannomas or neurilemmomas are benign, encapsulated, slow-growing mesenchymal neoplasms that arise from Schwann cells and have a low malignant potential. These neoplasms can present at any age, and most commonly occur in adult females between the ages of 20 and 50 years old. Up to 20% of cases are associated with neurofibromatosis type 1 (4). Although sporadic cases of these tumors arising in the retroperitoneum, pelvis, perineum, adrenals, kidneys and inguen have been previously reported (1), their occurrence in the abdominal wall is extremely rare and only a few of these cases have been reported (1,4–6).

The majority of schwannomas arise from the nerve sheath of large peripheral nerves, occurring at the level of or below the subcutaneous fat layer (7). Thus, the clinical signs and symptoms may vary according to the tumor location. The tumors may be asymptomatic and only found incidentally during examination. However, when they grow larger, they can put pressure on the surrounding large nerves (1), as found in the patient of the present study.

Radiological examinations, CT or magnetic resonance imaging (MRI) can provide important information about these tumors, including the tumor site, its characteristics and its associations with other tissues. The imaging features of schwannomas are associated with its two components: Antoni type A (cellular component) and Antoni type B (myxoid component). The former consists of spindle-shaped cells that are arranged compactly in interlacing fascicles or short bundles. Antoni B schwannomas are loosely composed of reticular fibers, cysts, vascellum and the Schwann cells (8). Accordingly, schwannomas usually present as a well-defined, homogeneous soft-tissue mass on conventional CT scan (9,10). The tumors show minimal or mild heterogeneous enhancement following the injection of contrast agent (9,10). In the present case, marked peripheral enhancement was demonstrated during the artery and venous phases. The progressive centripetal fill-in was then observed, and homogeneous enhancement occurred in the lag period. The CT imaging observations of the schwannoma in this study were similar with that of a hemangioma. In general, schwannomas appear as hypointense on T1-weighted and hyperintense on T2-weighted MRI. The hypercellular (Antoni A) and hypocellular (Antoni B) components display hypointense and hyperintense signals, respectively, on T2-weighted images (4,9). With gadolinium administration, the enhancement portion corresponds to the solid component of the Antoni A. Loose cellularity with diffuse edematous change may result in minimal contrast enhancement. If there is degenerative change of the neurilemmoma, it often shows a poor blood supply, cyst formation, calcifications, hemorrhage and hyalinization (11). In the present case, no cyst formation, calcification or necrosis was observed.

Although MRI and CT are ideally suited to detect the tumor pathology and delineate the soft tissue and its components, the final diagnosis can only be confirmed by histopathological examination. Immunohistochemical examinations besides hematoxylin and eosin staining are required to form a differential diagnosis (12). Although a previous study has shown that the diffuse immunoreactivity for S-100 is almost universal in schwannomas (13), another study showed that 10–50% of cases were negative (5). In the present case, the schwannoma was also negative for S-100.

The ideal treatment for schwannoma is complete surgical excision, and the prognosis is good (11), although recurrence of schwannomas *in situ* or at a distant site, attributed to incomplete resection, has also been reported (2). The present patient recovered well and no evidence of recurrence was found on CT images during the 3-month follow-up.

In conclusion, the present study indicates that a diagnosis of schwannoma should be considered for certain patients with masses in the abdominal wall. The observations of gradual and heterogeneous enhancement during the artery and venous phases, progressive centripetal fill-in and homogeneous enhancement during the delayed phase may be useful information for the imaging diagnosis of schwannomas.
